# Supplementary material for: Direct imaging of APP proteolysis in living cells
Source: PeerJ. 2017 Apr 12;5:e3086. doi: 10.7717/peerj.3086 (PMC5391788; doi:10.7717/peerj.3086)
Supplement: Supplemental Information 1 [file peerj-05-3086-s001.pdf]

Direct Imaging of APP Proteolysis in Living Cells

Niccoló Parenti, Ambra Del Grosso, Claudia Antoni, Marco Cecchini, Renato Corradetti, Francesco S. Pavone, Martino Calamai

Supplementary Information

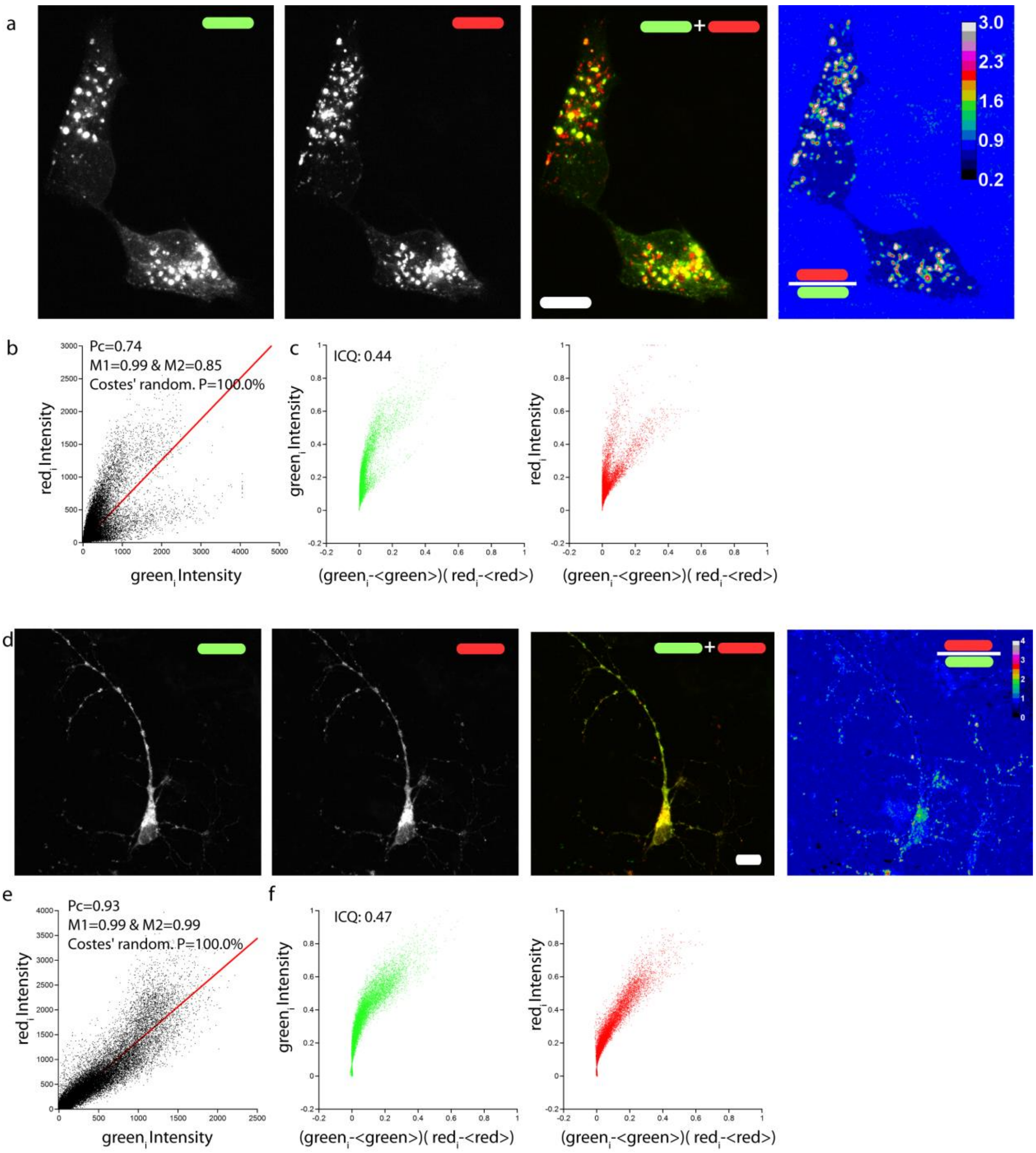

Supplementary Fig. S1 mChAPP770mGFP Red/green fluorescence intensity ratio. Maximum intensity projections of confocal z-stacks of living human SH-SY5Y cells (a-c) and rat hippocampal neurons (d-f) transfected with mChAPP770mGFP. The red/green ratio images have been mean filtered (2 pixels) only for representation purposes. The high degree of co-localisation of the red and green signals evident in the scatterplots (b-e) and the Li's intensity correlation analysis (c-f) is confirmed by the Pcs, M1s and M2s close to 1, and by the ICQs close to 0.5. The probability of obtaining the observed Pcs by chance is inversely correlated with the Costes' randomization P value, which in both cases is 100%. Scalebars, 10 µm.

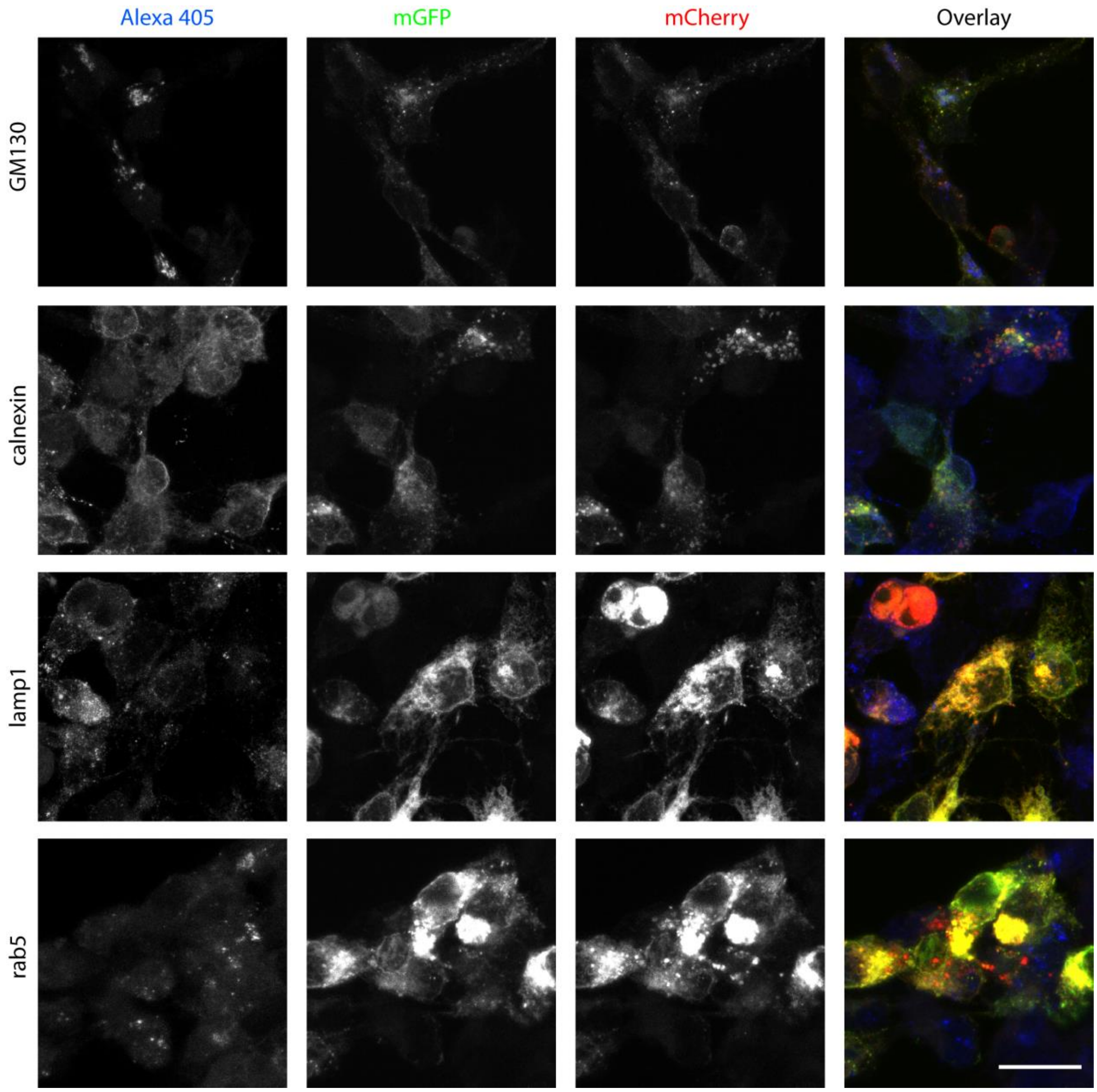

Supplementary Fig. S2 Subcellular localization of mChAPPmGFP. Maximum intensity projections of confocal z-stacks of fixed and permeabilized HEK cells transfected with mChAPP695mGFP and labeled with primary - anti-lamp1 ab24170 (lysosome marker), anti-rab5 ab18211 (endosome marker) and anti-calnexin ab22595 (endoplasmic reticulum marker) from Abcam, and anti-GM130 610822 (cis-Golgi marker) from BD Transduction laboratories - and secondary antibodies coupled to Alexa 405 (Life Tech-Thermofisher). Scalebar,10 µm.

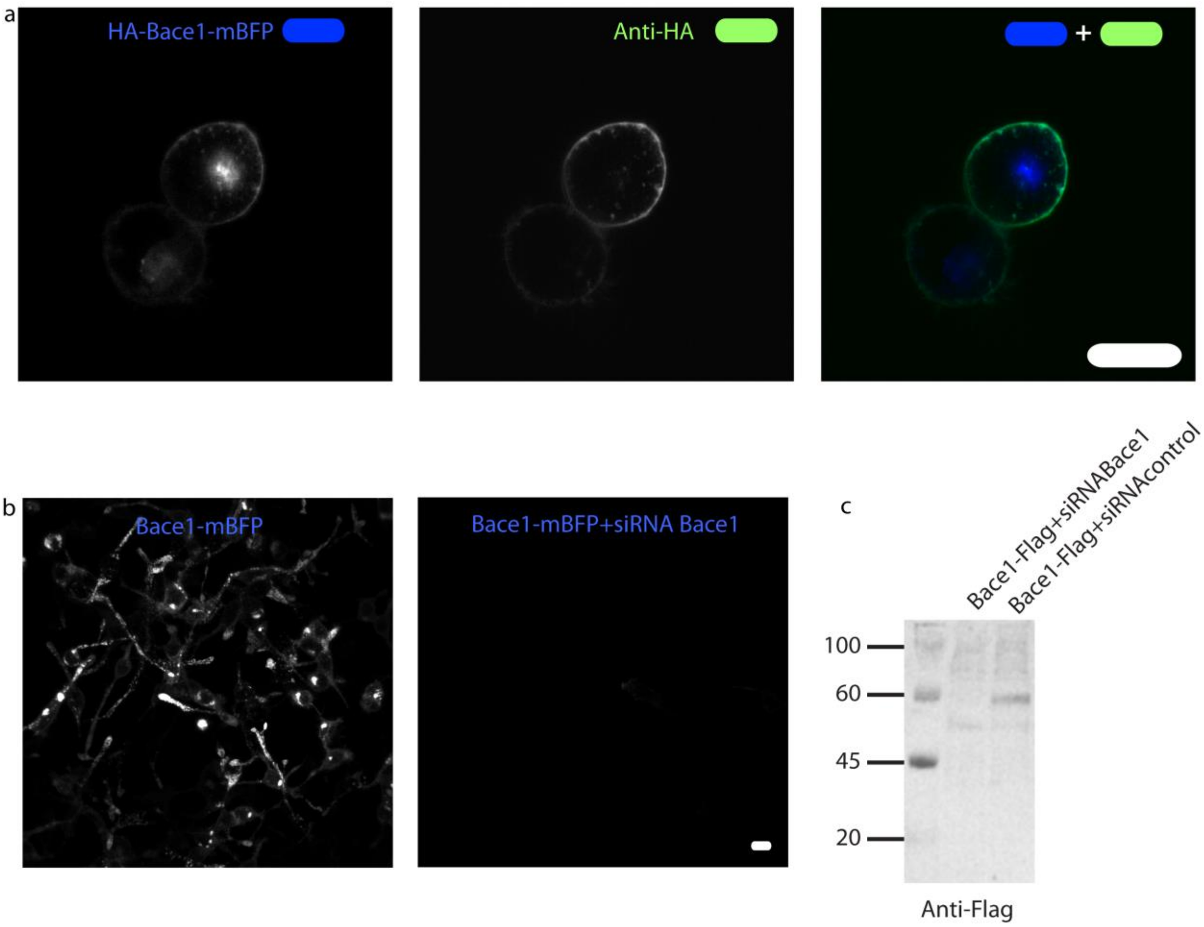

Supplementary Fig. S3 Overexpressed Bace1 distribution and siRNA test. (a) To verify the presence of Bace1 on the plasma membrane, we surface labeled HA-Bace1-mBFP with an anti-HA antibody coupled to secondary Alexa 488 antibody. To verify that Bace1-siRNA really affected the expression of Bace1, we have performed a test by transfecting SH-SY5Y cells with Bace1-mBFP (b), or Bace1-Flag (c), with or without Bace1-siRNA. The intensity of the blue fluorescence dramatically decreases in cells transfected with Bace1-mBFP and Bace1-siRNA (b), as well as the band around 57 kDa (predicted molecular weight of Bace1 is 56 kDa) revealed with anti-flag antibody disappears in cells transfected with Bace1-Flag and Bace1-siRNA (c) . Scalebar,10 µm.

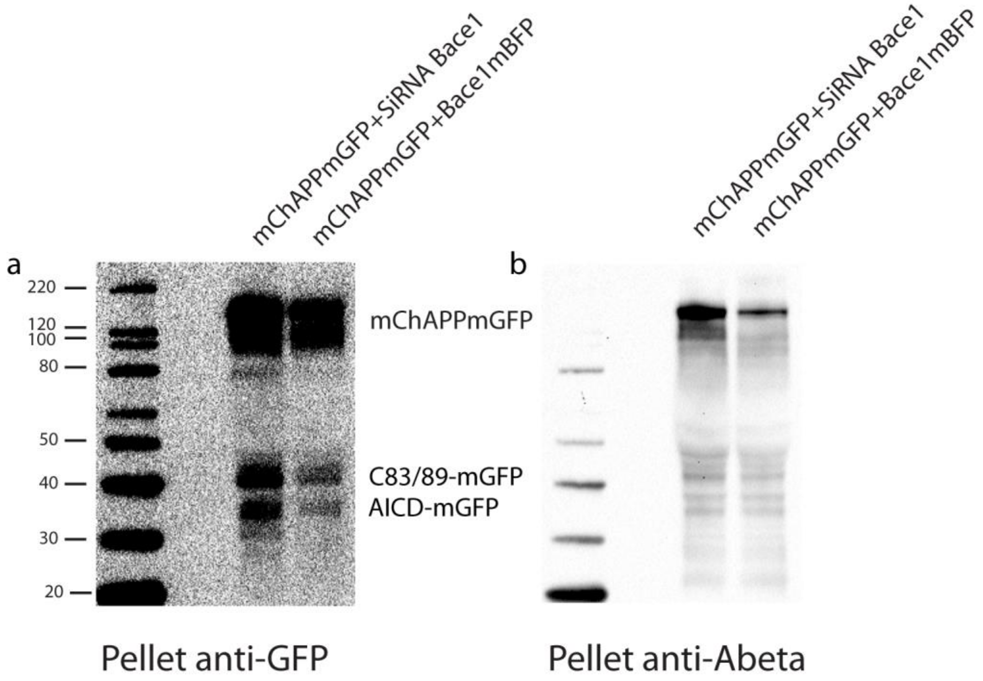

Supplementary Fig. S4 C-term fragments of mChAPPmGFP revealed with Westernblot. Immunoblots of human SH-SY5Y cells co-transfected with mChAPPmGFP and Bace1-mBFP. The cell lysates were labelled with anti-GFP (a), stripped and reprobed with anti-Aβ (b). The lower two bands at 40 and 35 kDa correspond to C83/89-mGFP and AICD-mGFP (expected Mw for mGFP = 27 kDa, C83/89 = 9-11 kDa, AICD = 5-7 kDa), supporting the appropriate processing of mChAPPmGFP. The blot using anti-Aβ antibody shows a higher degree of unspecific labeling. Although the bands are fainter when Bace1mBFP is co-expressed, the ratio between the amount of C83/89-mGFP, or AICD-mGFP, and full length mChAPPmGFP detected with the anti-GFP antibody results higher (~1.5 folds) than in the presence of siRNA against BACE1.

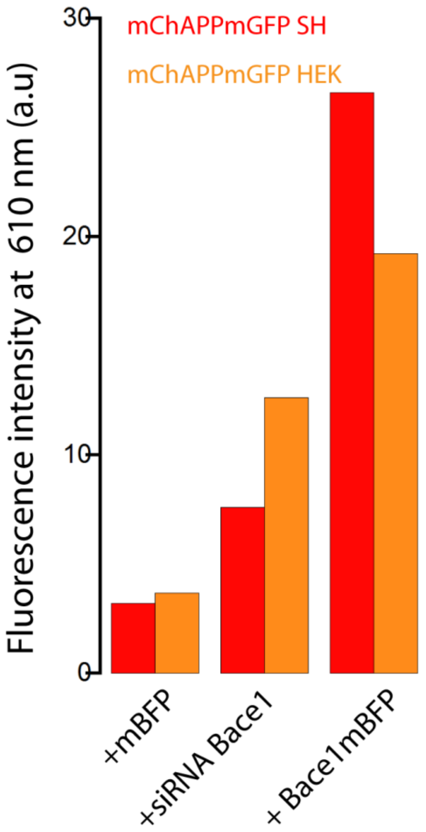

Supplementary Fig. S5 Fluorescence spectrophotometer. The fluorescence emission intensity peaks at 610 nm (following excitation at 550 nm) of the extracellular medium of HEK cells under different conditions of transfection were analogous to those of SH-SY5Y cells.

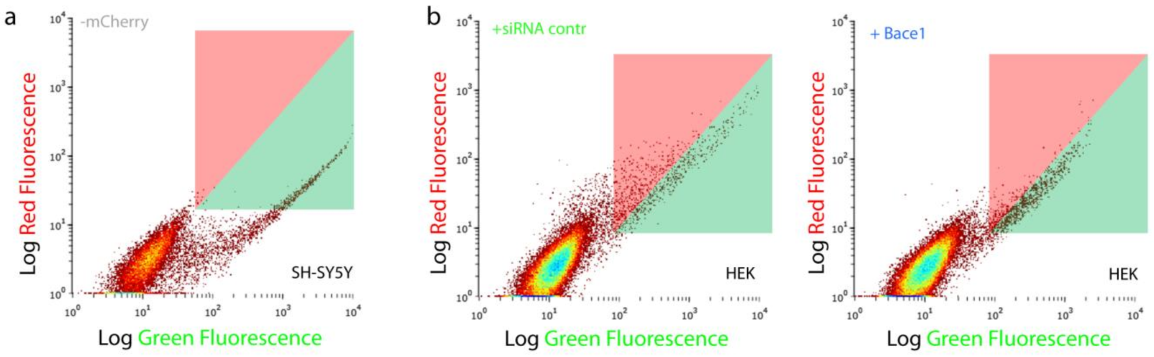

Supplementary Fig. S6 FACS flowcitometry of mChAPPmGFP in HEK cells. (a) Scatter plot of SH-SY5Y cells transfected with APPmGFP. (b) Scatter plot of HEK cells transfected with mChAPPmGFP and Bace1-mBF or control siRNA.

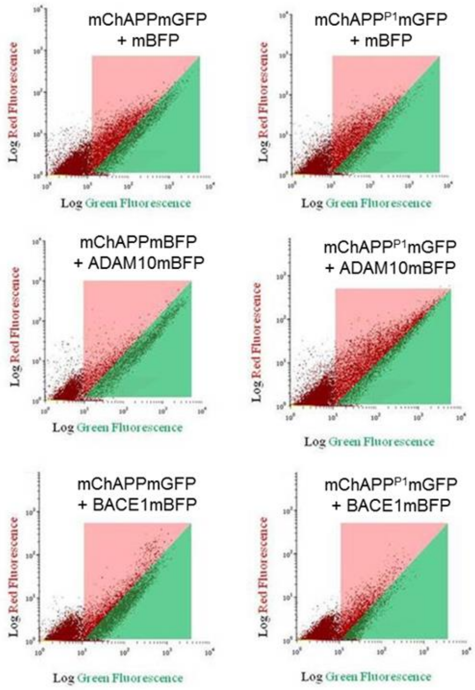

Supplementary Fig. S7 FACS flowcitometry of mChAPPmGFP and mChAPP^{P1}mGFP with ADAM10mBFP or BACE1mBFP. Scatter plots of SH-SY5Y cells transfected with mChAPPmGFP or mChAPP^{P1}mGFP together with mBFP, ADAM10mBFP or BACE1mBFP. Each dot in the scatter plot represents the green and red fluorescence intensity of a single cell. The two different areas (red and green) in the scatter plot were arbitrarily chosen so that both areas contained the same number of cells co-transfected with mChAPPmGFP and mBFP. These regions were used to gate the scatter plots of cells co-transfected with the other conditions and calculate the mean ratio between the number of cells in the red area over the number of cells in the green area.

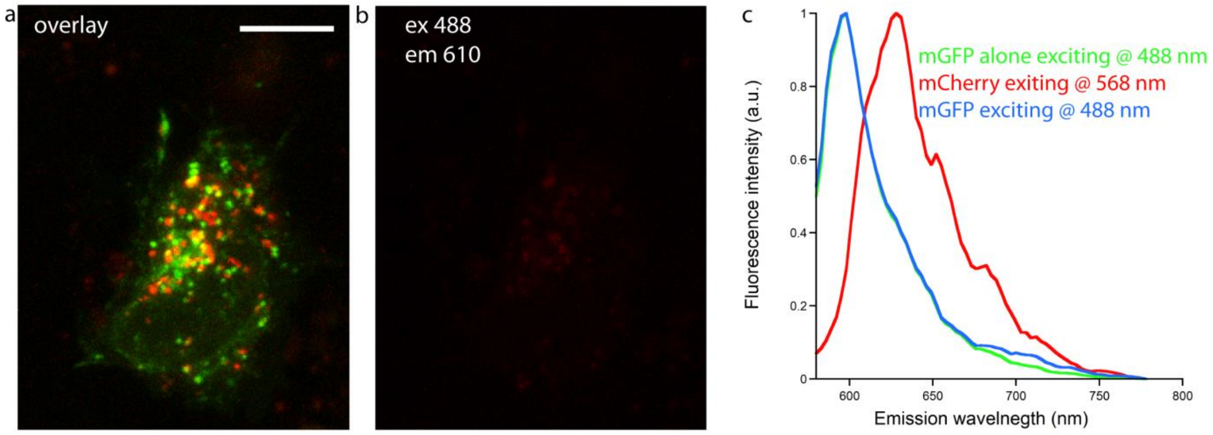

Supplementary Fig. S8 No significant FRET between mCherry and mGFP. When SH-SY5Y cells transfected with mChAPPmGFP (a) were excited using a 488 nm laser and imaged using a 610/20 nm (maximum fluorescence emission of mCherry) bandpass filter, the fluorescence intensity was not significantly different from that of the background (b). Moreover, we analyzed the fluorescence emission spectra of both mGFP and mCherry in different regions of the cell body. We did not find any alteration in the mGFP emission spectra due to the mCherry component, confirming the very weak FRET efficiency (c).
